# The prevalence, reasons and attitudes for the practice of informal medicine

**DOI:** 10.1186/s12875-020-01362-z

**Published:** 2021-02-03

**Authors:** Menashe Meni Amran, Avital Bilitzky Kopit, Hannan Ariel Kranc, Roni Peleg

**Affiliations:** 1 Maccabi Health Services, Tel Aviv, Israel; 2 Ben Gurion University of the Negev, Be’er Sheva, Israel; 3 Clalit Health Services, Tel Aviv, Israel

**Keywords:** Informal medicine, Informal consulting, Primary care, Family medicine

## Abstract

**Background:**

Informal medicine, entailing undocumented medical advice, has been described in diverse medical disciplines and geographical regions. We assessed the current prevalence and characteristics of informal medical consulting, the reasons physicians provide it, and their attitudes toward it.

**Methods:**

We conducted a survey among family physicians in Israel, a country with a national health insurance system. A questionnaire was emailed to all primary care physicians in the two largest HMOs in southern Israel. Fifteen questions addressed the prevalence, practice and attitudes to informal medical consulting. Ten questions assessed demographics and professional experience.

**Results:**

Of 143 respondents (mean age 41 years), 55% were women. Ninety-five percent of the respondents reported requesting informal medical consulting from other physicians. Fifty-four percent reported often providing informal consulting to family and friends; and an additional 27% reported doing so under exceptional circumstances. The main reasons given for informal consulting requests were availability and accessibility (81% of respondents), and not financial savings. Only 17.5% stated being in favor of informal consulting for family and friends. Only 11% expressed feeling satisfaction after providing such consultation; 49% expressed discomfort. Sixty-six percent thought a position paper on informal consulting to family and friends is needed.

**Conclusions:**

Our survey of primary care physicians shows very frequent informal medical consulting, despite high dissatisfaction with such, and an interest in receiving guidelines on this practice.

**Supplementary Information:**

The online version contains supplementary material available at 10.1186/s12875-020-01362-z.

## Introduction

Informal medical consultation, in contrast to formal medical consultation, is characterized by the provision of undocumented medical advice. This includes any medical consultation or treatment provided to colleagues, family members or friends. Numerous surveys and editorials have described the intervention of physicians in the health care of family members [1]. The practice has persisted over the years, despite the inherent problems and the recommendations by such publications and by medical associations against it. In addition to family members, friends and persons of other relations request and receive informal medical consultation. Described as “corridor”, “hallway” or “curbside” consultation, such practice has been described in diverse medical disciplines [2, 3] and geographical regions [1, 4] Self-care and self-prescribing by physicians have also been widely described [5–10].

Israel has a national health insurance system, in which all the residents are insured by one of four health maintenance organizations (HMOs). Family physician visits are at no cost. Small co-payments are charged for visits with specialists, though low socioeconomic status exempts also from these costs. During 2016, nearly 83% of Israeli households purchased complementary health insurance from their HMOs [11]. The benefits of such include subsidies for second opinion consultations and scheduling appointments with shorter waiting periods.

In a survey study conducted in Israel over 20 years ago, 82% of hospital physicians reported having been asked to provide “hallway medicine”; of them, 91% agreed [12]. However, no position paper has been issued in Israel over the last two decades to guide physicians in dealing with this phenomenon. We conducted a survey among family physicians in southern Israel, to assess the current prevalence and characteristics of informal medical consulting, the reasons physicians provide it, and their attitudes toward it.

## Methods

This multicenter survey study is based on questions written by the researchers. The study population is primary care physicians (family physicians, general physicians and residents) who are employed in southern Israel. In total, 595 physicians were eligible to participate, from the two largest HMOs in Israel: 356 from Clalit Healthcare Services and 239 from Maccabi Health Services.

The questionnaire was designed to access information regarding the prevalence, reasons, means of practice and attitudes to informal medicine consultation among primary care physicians. A pilot test was performed on the initial questionnaire, among 10 participants. Following their comments, the questionnaire was revised to the final version (see Additional file 1). The questionnaire comprised 15 questions on practice and attitudes; each with 2–6 possible responses. One question asked the extent that the physicians consider each of 5 factors when approached for informal advice; the responses were on a 5-point Likert scale. Ten questions accessed information on demographics and professional experience.

The questionnaires were sent by a link to all the email addresses of the primary care physicians affiliated with Maccabi Healthcare Services and Clalit Health Services in the southern district of Israel. A request was included on the questionnaires, that physicians should not fill the questionnaire more than once. Three reminders were sent, at intervals of 3–4 weeks.

### Statistical analysis

Statistical analysis was performed using the IBM SPSS version 25. Data were reported as means and standard deviations for continuous variables, and as percentages for categorical variables. We used the Student’s t-test to determine statistically significant differences in continuous variables that were normally distributed. For continuous variables that were not normally distributed or ordinal variables, we used the Mann-Whitney test or Kruskal–Wallis test, as appropriate. The Chi-square test and Fisher’s test were used to compare categorical variables. All *p*-values were two-sided and statistical significance was set at *P* ≤ 0.05.

The protocol was approved by the institutional review board of Maccabi Healthcare Services and the ethical committees of Maccabi and Clalit Health Services.

## Results

### Study population

The total number of respondents was 143, for a response rate of 24%. Table 1 presents the self-reported demographic characteristics of the respondents. The majority of the respondents were women, 55%. The mean age was 41 years. The majority of respondents work primarily in urban clinics, 62%. Half of those who stated their specialty were family medicine specialists; 28.5% were residents. Forty-one percent of the respondents had less than 5 years seniority; 30% had more than ten years.

**Table 1:** Characteristics of the respondent physicians (*N* = 143)

Characteristics	Respondent physicians	Non-responders
Gender, % (n)		
Men	44.6% (62)	4
Women	55.4% (77)	
Age, mean ± SD	41.3 ± 8.8	14
Familial status, % (n)		4
Single	7.9% (11)	
Married	89.2% (124)	
Divorced	2.9% (4)	
Origin, % (n)		13
Israel	70.8% (92)	
Elsewhere	29.2% (38)	
University, % (n)		15
Israel	69.5% (89)	
Elsewhere	30.5% (39)	
Workplace, % (n)		4
Urban primary care clinic	61.9% (86)	
Rural primary care clinic	24.5% (34)	
Public hospital	5.8% (8)	
Combination	7.9% (11)	
Education level / Specialization % (n)		6
General practitioner without specialization, %	15.3% (21)	
Resident	28.5% (39)	
Specialist in family medicine	50.4% (69)	
Specialist in internal medicine	5.8% (8)	
Seniority, median (range)	5 (0.5–45)	15
Less than 5 years, % (n)	41.4% (53)	
6–10 years, % (n)	28.9% (37)	
More than 10 years, % (n)	29.7% (38)	

“Specialists” include specialist in family medicine and specialist in internal medicine

“Non-specialists” include resident and general practitioner without specialization

Table 2 presents the responses to the questions regarding informal medical consulting.

**Table 2:** Responses to the questionnaire

Question	Number	Percent
**1. Did you at any time request informal medical consulting from another doctor?**		
Yes	135	94.4%
No	8	5.6%
	143	
**2. During the last month, how often did people turn to you for informal medical consulting?**		
A number of times a day /Once a day	41	28.9%
2–3 times a week/Once a week or less	91	64.1%
Not at all	10	7.0%
	142	(mis = 1)
**3. By what means were the requests for informal medical consults directed to you (more than one response can be selected)?**		
Face-to-face meeting, planned in advance	121	84.6%
Incidental meeting such as a social event	99	69.2%
Phone messages (such as Whatsapp)	103	72.0%
Electronical mail	114	79.7%
Other	11	7.7%
	143	
**4. To what degree are you in favor of formal medical consulting for family members and friends?**		
Strongly in favor/In favor	38	26.6%
Neutral reaction	41	28.7%
Not in favor/Strongly opposed	64	44.8%
	143	
**5. To what degree are you in favor of informal medical consulting for family members and friends?**		
Strongly in favor/In favor	25	17.7%
Neutral reaction	51	36.2%
Not in favor/Strongly opposed	65	46.1%
	141	(mis = 2)
**6. Do you give informal consulting to family and friends?**		
Yes, often	77	53.8%
Yes, under exceptional circumstances	39	27.3%
I try to avoid it/Never	27	18.9%
	143	
**7. If you answered yes on the previous question, what type of consulting to you provide to family members and friends (more than one response can be selected)?**		
Referrals to the emergency room	66	46.5%
Recommendation to medical specialists	96	67.6%
Prescriptions for drugs	98	69.0%
Interpretation of results of medical testing (blood tests, imaging, etc)	115	81.0%
Routine examinations	31	21.8%
Treatment in emergency situations	72	50.7%
Requests for a second opinion	62	43.7%
	142	(mis = 1)
**8. How would you describe your feeling after providing such consultation?**		
Satisfaction	16	11.3%
Indifference	34	23.9%
Discomfort	70	49.3%
Regret	12	8.5%
Other	10	7.0%
	142	(mis = 1)
**9. What do you think are the main reasons that people turn to you informally rather than to their family physician?**		
Savings in treatment costs	3	2.2%
Accessibility and availability	116	83.5%
Lack of trust in the public healthcare system	16	11.5%
Confidentiality	4	2.9%
	139	(mis = 4)
**10. Did you ever refuse a request for informal consulting from a family member or a friend?**		
Yes, always/Yes, most often	10	7.0%
Sometimes	65	45.5%
Usually not/ Never	68	47.6%
	143	
**12. Did you every receive compensation (financial or other benefits) for medical treatment or from preferring informal medicine for a family member or friend?**		
Yes	22	15.4%
No	121	84.6%
	143	
**13. What do you think is the disadvantage of informal medicine (more than one response can be selected)?**		
A lack of medical documentation	121	85.2%
Lack of the patient’s full consent	30	21.1%
Lack of objectivity	88	62.0%
The risk of unprofessionalism or negligence	85	59.9%
There are no particular disadvantages	5	3.5%
	142	(mis = 1)
**14. Have you provided informal medicine by telephone or by text messaging?**		
Yes	137	95.8%
No	6	4.2%
	143	
**15. If you answered yes on the last question, what is your opinion regarding such?**		
It’s legitimate/It’s not ideal, but adequate in certain situations	72	51.1%
It’s only suitable for emergency situations	31	22.0%
It’s problematic and best to avoid	38	27.0%
	141	(mis = 2)
**16. No position paper exists at this time of an ethical committee regarding the provision of informal medicine to family members and friends. Do you think such position paper is needed?**		
Yes	94	66.2%
No	48	33.8%
	142	(mis = 1)

### Frequency of informal medical consulting

**Fig. 1:**
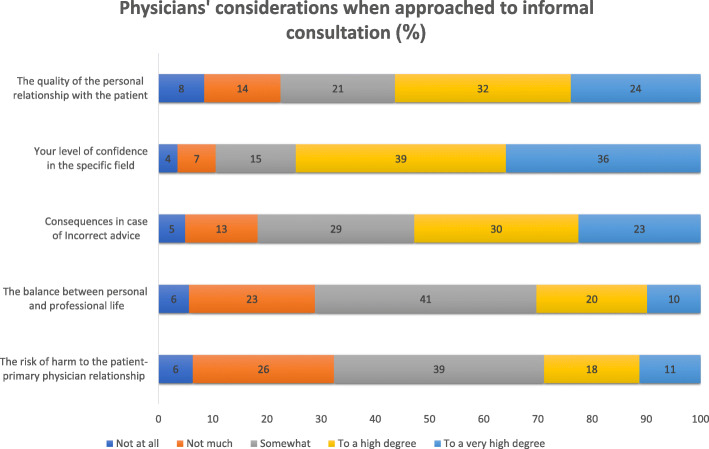
Physicians’ considerations when approached to informal consultation (Question 11). The numbers represent percentages as follows: the green colour represents the percentage of physicians who graded the specific consideration as ‘very high level’, the yellow colour represents ‘high level’, grey colour ‘medium level’, orange colour ‘low level’ and red colour ‘not at all’. Total *N* = 142

The vast majority, 95%, of the respondents reported requesting informal medical consulting from other physicians (Question 1). Thirty percent provided such service at least once daily, during the preceding month (Question 2). Only 7% reported not providing any such consulting over the last month. Fifty-four percent of the respondents reported that they often provide informal consulting to family and friends, and an additional 27% reported doing so under exceptional circumstances (Question 6). Forty-eight percent answered that they usually accept or never refused a request for informal medical consulting from family and friends (Question 10). Seventy-five percent of the respondents stated that they highly or very highly considered their confidence in the field, when approached for informal consultation (Question 11, Fig. 1). Fifty-six percent responded that they highly or very highly considered the quality of their personal relation with the individual requesting the consultation, and 53% reported considering highly or very highly the consequences of providing incorrect advice.

### Characteristics of informal medical consulting

**Fig. 2:**
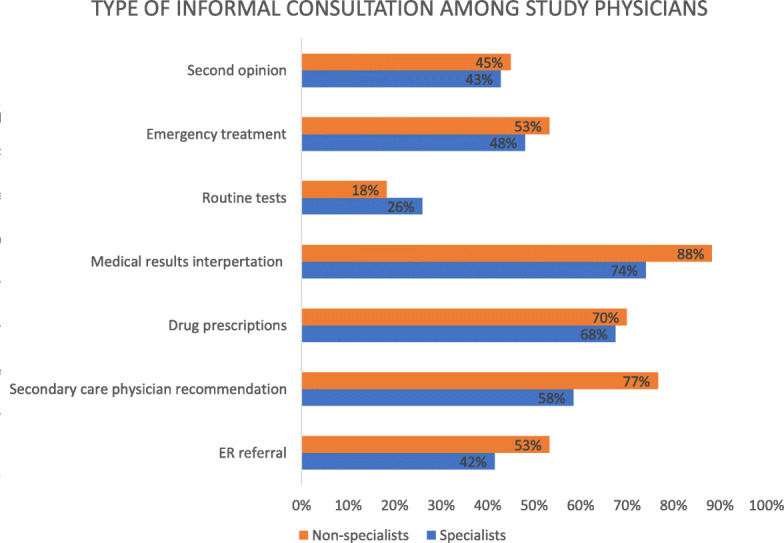
Type of informal consultation among study physicians (Question 7). The orange bar represents the percentages of residents and general practitioners without specialization (“non-specialists”) who reported providing each type of informal consultation. The blue bar represents the corresponding percentages among specialists (Include family medicine and internal medicine specialists). Differences were statistically significant regarding “medical results interpretation” (*P* = 0.037) and “secondary care physician recommendation” (*P* = 0.025). *P*-value was calculated using Chi-square test. ER: emergency room

Eighty-five percent of the respondents reported providing informal consulting face-to-face. In addition, high proportions reported also providing consultation by means of phone messages such as WhatsApp Messenger, and by electronic mail. (Question 3). The vast majority, 96%, reported providing informal consulting by phone (Question 14). More than half the respondents reported providing consultation in non-emergency situations, such as interpreting medical tests or providing routine examinations (Question 7). Only 5% of responders reported avoiding medical treatment and providing only clarification, interpretation or summation of clinical information, such as the recommendation of a specialist or the interpretation of medical. Residents and general practitioners (“non-specialists”) more frequently reported interpreting medical results and recommending secondary care physicians than did specialists in internal medicine and family medicine (“specialists”) (Fig. 2). The main reason presumed for the requests for informal consulting were availability and accessibility, as selected by 81% of the respondents (Question 9). Fifteen percent of the respondents reported receiving some form of compensation for providing informal medical consulting to family or friends (Question 12). No differences were found between specialists and residents in the proportions that reported refusing to provide informal consulting, and no association was found between seniority and refusing to provide consulting.

### Attitudes to informal medical consulting

Only 27% responded that they were in favor of formal medical consulting for family and friends (Question 4); and only 17.5% responded being in favor of informal consulting for family and friends (Question 5). Respondents who reported receiving more requests for informal consults expressed more opposition to this type of consulting (*p* = 0.048). However, those who provided more informal consultations to family and friends expressed their support of doing such (*p* = 0.012), and their feelings following these consultations were more positive (*p* < 0.001).

**Fig. 3:**
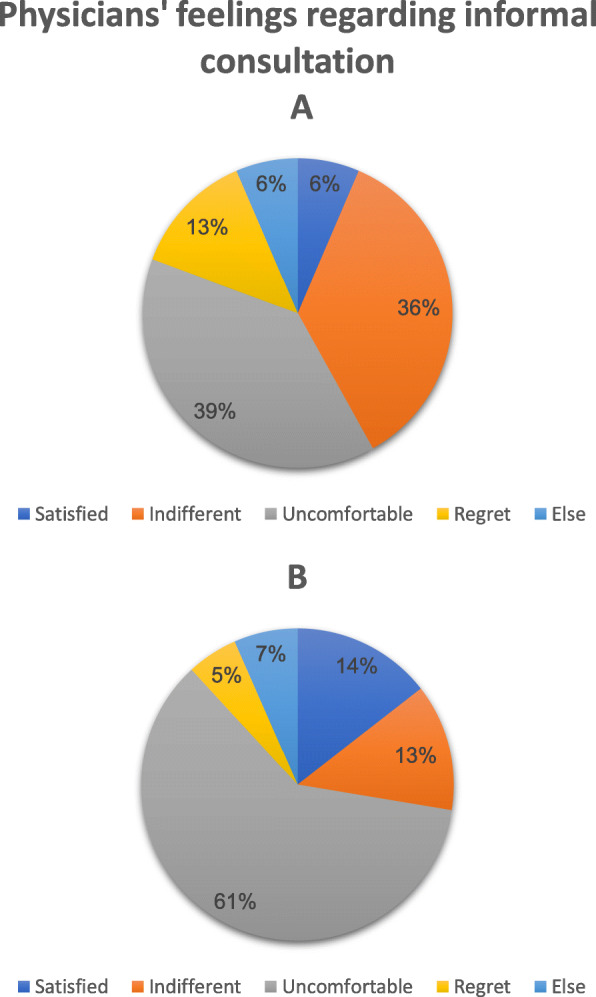
Physicians’ feelings regarding informal consultation (Question 8). Pie A demonstrates feelings among men while pie B demonstrates feelings among women. Male physicians expressed more indifference regarding informal consultation compared to female physicians (*P* = 0.002), as calculated using the Chi-square test

Only 11% of the respondents expressed feeling satisfaction after providing informal consultation; almost half, 49%, expressed discomfort (Question 8). Differences were observed between men and women in their feelings after providing consultation (Fig. 3). Sixty-one percent of the women compared to 39% of the men expressed feeling uncomfortable. Thirty-six percent of the men compared to 13% of the women felt indifferent (*P* = 0.002). Of those who reported providing consultation by phone (96%), almost half (49%) answered that such means should only be used under emergency situations or should be avoided (Question 15). The main disadvantage to informal medical consulting according to the respondents was the lack of medical documentation, as cited by 85%. Lack of objectivity and the risk of unprofessionalism or negligence were also cited by the majority of respondents (Question 13).

Sixty-six percent of the respondents thought that a position paper on informal consulting to family and friends is needed (Question 16). Such position paper was more often desired among those who expressed having negative rather than positive or indifferent feelings after providing informal consulting (*P* = 0.002); and also among those who reported more than one daily request for informal consulting (*P* = 0.009).

## Discussion

This survey study revealed great dissonance among primary care physicians, between their behavior and their attitudes, in regard to informal medical consulting. On one hand, an overwhelming proportion reported involvement in such consulting, including more than half who reported frequent rates. Further, almost half reported that they usually or never refuse a request from family or friends for informal consulting. On the other hand, more than half the participants in the survey stated feeling discomfort or regret after providing informal consulting. More specific analysis of the data reveals that some respondents may resolve the dissonance integral to their provision of informal medical consulting. Specifically, those who reported more frequently providing informal consulting, expressed greater support of such, and their feelings following informal consultations were more positive than were those who less frequently provided such consultations.

The high proportion of physicians reporting informal consulting concurs with other studies, most of which focused on consulting to family members [1]. Nonetheless, the report by 30% of family physicians in the current survey, of providing informal consulting on a daily basis is remarkable. A particularly high prevalence of informal medicine in Israel may be related to cultural factors. Along this line, the practice in Israel of informal payments for health care has been explained in the context of a specific type of political culture, called “alternative politics” [13]. This is characterized by a “do-it-yourself” approach, which bypasses formal rules and relies on personal and reciprocal relations. The scope of this approach is broad, and may contribute to understanding the atmosphere that makes it difficult for physicians to refuse requests for informal consulting [12].

The negative attitude toward informal medical consulting expressed by the respondents of the current survey corroborates other publications [1]. Problems related to the lack in medical documentation, objectivity, and professionalism were the main disadvantages cited for informal medical consultation, concurring with the literature [1].

Only 2% of the respondents presumed that financial savings was the motivation for informal consulting. This may reflect a pervasive impact of the national health insurance system in Israel, despite the heavy reliance of the health care system on private financing [11]. In contrast, among 41 studies, financial savings was cited as a main reason for the intervention of physicians in the health care of family members [1].

In the current investigation of informal consultation, the high use reported of electronic mail and phone messages, including WhatsApp Messenger, is in agreement with the currently high use of these means of communication in formal medical consultation. WhatsApp Messenger has become a common telemedicine tool in conventional, as well as in informal medicine [14]. In a study conducted among primary care physicians in Switzerland, 82% reported communicating with their patients by email [15]. The authors emphasized confidentiality issues as a prime disadvantage to such.

Ninety-five percent of our respondents reported requesting informal consultation from other physicians for their personal health issues. This corroborates the documentations of this phenomenon around the world, as mentioned above. Notably, a recently published cross-sectional study showed that two-thirds of hospital-based physicians in Israel do not have a regular personal physician [16].

Almost two-thirds of the respondents to our survey answered that a position paper on informal medical consulting could be beneficial. The proportion holding this attitude was particularly high among physicians who had more negative feelings after providing informal consultation and among those who reported receiving more than one daily request for informal consulting. The seventh edition of the American College of Physicians Ethics Manual, issued in 2019, [17] expanded the topic of informal medical consulting, as well as the topics on electronic communication and telemedicine ethics. Accordingly, physicians are encouraged to avoid treating themselves and family members except in emergency situations. Among the reasons cited earlier by the American Medical Association for such recommendation are difficulties in objectivity, in accessing full information and in professionalism that arise in the context of informal medical consulting [18]. Our study considered informal medicine in a broader sense than in the American College of Physicians Ethics Manual. Remarkably, 95% of our responders reported providing informal medicine in the form of treatment and health care management, and not only clarification and interpretation of clinical information. More detailed guidelines may be beneficial to physicians, with a broader scope in regard to the nature of informal consulting, and including consulting of persons who are not family members.

No differences were found between specialists and residents in the responses to any of the items of the survey. This contrasts with the findings of a qualitative study conducted in the Netherlands, which showed more difficulties among junior than senior physicians in dealing with requests for informal consulting [19].

A main limitation of this survey study is selection bias, arising from the possibility that the respondents to the survey may not have been representative of the family physicians in the region examined. The questionnaire was kept short, so as to encourage respondents to fill it completely. Accordingly, very few responses were left blank. Nonetheless, the brief and structured questionnaire is limited by the information it was able to assess, compared to a more in-depth questionnaire or an interview.

## Conclusions

According to a survey of family care physicians in Israel, the vast majority provide informal medical consulting to family and friends, a high proportion of them do so frequently. Discomfort and regret following such consultations were reported among many. Interest was expressed in receiving guiding principles on the matter. Due to the cultural influences inherent to informal medical consulting, more studies and specific guidelines in different geographical regions may help elucidate the problem and its consequences in various contexts. Overall, physicians seem to need more guidance and tools to help them say “No” when this is the ethical and professional response.

## Supplementary Information

**Additional file 1.**

## Data Availability

The datasets used and/or analysed during the current study are available from the corresponding author on reasonable request.
