# Supplementary material for: The prevalence, reasons and attitudes for the practice of informal medicine
Source: BMC Fam Pract. 2021 Feb 3;22:30. doi: 10.1186/s12875-020-01362-z (PMC7860190; doi:10.1186/s12875-020-01362-z)
Supplement: Supplementary file 1 — Additional file 1. [file 12875_2020_1362_MOESM1_ESM.pdf]

The prevalence, reasons and attitudes for the practice of informal medicine

Menashe Meni AMRAN*[1, 2], Dr Avital Bilitzky KOPIT[1], Hannan Ariel KRANC[2], Roni PELEG[2,3]

[1]Maccabi Health Services, Tel Aviv, Israel

[2]Ben Gurion University of the Negev, Be’er Sheva, Israel

[3]Clalit Health Services, Tel Aviv, Israel

APPENDIX. The study questionnaire

Dear Doctor,
We are conducting research on informal medicine among primary care physicians. Informal medicine comprises medical consulting (including prescribing medications, referrals, and diagnoses) that are not documented in the medical records of the patient, whether or the consulting is paid or unpaid.

This research is being conducted in the framework of research practice in the department of family medicine. All the responses will be kept discrete and will be used for research purposes only.

You may choose not to answer any question in the questionnaire or on some of them, and to stop at any stage. We assure that your personal identify will be kept confidential by all those involved in this research.

Please do not fill the questionnaire if you did so in the past.

We appreciate your cooperation.

1. Did you at any time request informal medical consulting from another doctor?
    a. Yes
    b. No
2. During the last month, how often did people turn to you for informal medical consulting?
    a. A number of times a day
    b. Once a day
    c. 2-3 times a week
    d. Once a week or less
    e. Not at all
3. By what means were the requests for informal medical consults directed to you (more than one response can be selected)?
    a. Face-to-face meeting, planned in advance
    b. Incidental meeting such as a social event
    c. Phone messages (such as Whatsapp)
    d. Electronical mail
    e. Other_____________
4. To what degree are you in favor of ***formal*** medical consulting for family members and friends?
    a. Strongly in favor
    b. In favor
    c. Neutral reaction
    d. Not in favor
    e. Strongly opposed
5. To what degree are you in favor of ***informal*** medical consulting for family members and friends?
    a. Strongly in favor
    b. In favor
    c. Neutral reaction
    d. Not in favor
    e. Strongly opposed

6. Do you give informal consulting to family and friends?
    a. Yes, often
    b. Yes, under exceptional circumstances
    c. I try to avoid it
    d. Never
7. If you answered yes on the previous question, what type of consulting to you provide to family members and friends (more than one response can be selected)?
    a. Referrals to the emergency room
    b. Recommendation to medical specialists
    c. Prescriptions for drugs
    d. Interpretation of results of medical testing (blood tests, imaging, etc)
    e. Routine examinations
    f. Treatment in emergency situations
    g. Requests for a second opinion
8. How would you describe your feeling after providing such consultation?
    a. Satisfaction
    b. Indifference
    c. Discomfort
    d. Regret
    e. Other___________
9. What do you think are the main reasons that people turn to you informally rather than to their family physician?
    a. Savings in treatment costs
    b. Accessibility and availability
    c. Lack of trust in the public healthcare system
    d. Confidentiality
    e. Other____________
10. Did you ever refuse a request for informal consulting from a family member or a friend?
    a. Yes, always
    b. Yes, most often
    c. Sometimes
    d. Usually not
    e. Never

11. To what extent do you consider each of the following items when you are approached for informal advice?

	Not at all	Not much	Somewhat	To a high degree	To a very high degree
The quality of the personal relationship with the patient	a	b	c	d	e
Your level of confidence in the specific field	a	b	c	d	e
Consequences in case of Incorrect advice	a	b	c	d	e
The balance between personal and professional life	a	b	c	d	e
The risk of harm to the patient-primary physician relationship	a	b	c	d	e

12. Did you every receive compensation (financial or other benefits) for medical treatment or from preferring informal medicine for a family member or friend?
    a. Yes
    b. No

13. What do you think is the disadvantage of informal medicine (more than one response can be selected)?
    a. A lack of medical documentation
    b. Lack of the patient’s full consent
    c. Lack of objectivity
    d. The risk of unprofessionalism or negligence
    e. There are no particular disadvantages
    f. Other____________
14. Have you provided informal medicine by telephone or by text messaging?
    a. Yes
    b. No
15. If you answered yes on the last question, what is your opinion regarding such?
    a. It’s legitimate
    b. It’s not ideal, but adequate in certain situations
    c. It’s only suitable for emergency situations
    d. It’s problematic and best to avoid
16. No position paper exists at this time of an ethical committee regarding the provision of informal medicine to family members and friends. Do you think such position paper is needed?
    a. Yes
    b. No

We would appreciate your answering a number of demographic questions

17. Gender
    a. Male
    b. Female
18. Year of birth_____________
19. Family status
    a. Single
    b. Married
    c. Divorced
    d. Widowed
    e. Other
20. Country of birth
    a. Israel
    b. Other_________________ Year of immigration___________
21. Country of medical education
    a. Israel
    b. Other____________
22. The year of completion of medical school___________
23. The number of years working in the clinic__________
24. The type of your main clinic
    a. Urban
    b. Rural / suburban
    c. Hospital
    d. Combination
25. Your medical status
    a. General physician
    b. Resident

c. Family physician specialist
d. Internal medicine specialist
e. Other

26. Your affiliation with a health maintenance organization
    a. Clalit
    b. Maccabi
